# Long-Term Electrophysiological and Behavioral Analysis on the Improvement of Visual Working Memory Load, Training Gains, and Transfer Benefits

**DOI:** 10.4236/jbbs.2014.45025

**Published:** 2014-05-01

**Authors:** Ching-Chang Kuo, Cheng Zhang, Robert A. Rissman, Alan W. L. Chiu

**Affiliations:** 1 NeuroInformatics Center, University of Oregon, Eugene, USA; 2 Department of Biomedical Engineering, Louisiana Tech University, Ruston, USA; 3 Department of Neurosciences, University of California, San Diego School of Medicine, La Jolla, USA; 4 Applied Biology and Biomedical Engineering, Rose-Hulman Institute of Technology, Terre Haute, USA

**Keywords:** Visual Working Memory, Event-Related Potential, Cognitive Training

## Abstract

Recent evidence demonstrates that with training, one can enhance visual working memory (VWM) capacity and attention over time in the near transfer tasks. Not only do these studies reveal the characteristics of VWM load and the influences of training, they may also provide insights into developing effective rehabilitation for patients with VWM deficiencies. However, few studies have investigated VWM over extended periods of time and evaluated transfer benefits on non-trained tasks. Here, we combined behavioral and electroencephalographical approaches to investigate VWM load, training gains, and transfer benefits. Our results reveal that VWM capacity is directly correlated to the difference of event-related potential waveforms. In particular, the “magic number 4” can be observed through the contralateral delay amplitude and the average capacity is 3.25-item over 15 participants. Furthermore, our findings indicate that VWM capacity can be improved through training; and after training exercises, participants from the training group are able to dramatically improve their performance. Likewise, the training effects on non-trained tasks can also be observed at the 12th week after training. Therefore, we conclude that participants can benefit from training gains, and augmented VWM capacity sustained over long periods of time on specific variety of tasks.

## 1. Introduction

Visual working memory (VWM), or visual short-term memory, refers to a limited amount of information storage within few seconds [1], which is associated with important cognitive modalities, including attention, perception, reasoning, comprehension, and language acquisition [2] [3]. VWM also plays a critical role in preserving and processing information, and its capacity has been suggested to be a sensitive predictor of cognitive ability [4]. For example, researchers have implicated that VWM capacity can distinguish healthy or memory-impaired individuals suffering from attention-deficit hyperactivity disorder (ADHD) [5], schizophrenia [6], stroke [7], Alzheimer’s Disease [8]-[11], or age-related diseases associated with memory deficits [12] [13]. Recent evidence demonstrates that brain training can enhance an individual’s VWM capacity and attention over time [12] by increasing activity in the prefrontal cortex, the parietal cortex, and the basal ganglia [14] [15]. Not only do these studies reveal characteristics of VWM load and effects of training, they may also provide insights into effective rehabilitation means for patients with low VWM capacity. Furthermore, healthy individuals who seek to enhance their intellectual performance may also benefit from the training [14].

Despite potential applications of VWM, very few studies have investigated VWM over extended periods (*i.e*. beyond 5-weeks) and further evaluated transfer benefits on non-trained tasks [14]. Most of the research has been focused on distinguishing different memory systems and memory-processing phases to have a better understanding of memory characteristics and functions [16]. Considering this information is important correlated to VWM training, we employed a combined behavioral and electrophysiological test to reveal the impact of VWM load, training, and transfer effects on memory capacities and task-related performances. Event-related potentials (ERP) as the result of VWM information processing were recorded. Arrays of colored squares were used to estimate the VWM capacity through computerized tasks, broken into several experimental blocks. In addition, three features (color, position, shape) from tasks were taken into account in order to establish a complete model of VWM capacity.

The goal of this study is to evaluate the changes of the neural activity that accompany the training to perform a specific variety of tasks. We hypothesized that participants would exhibit increased neural activities in raising attention and memorization as a result of training paradigms to improve VWM. Three major experiments were conducted: (1) VWM load experiments. We estimated VWM capacity behaviorally through accuracy measurements and electrophysiologically through the amplitude level of contralateral delay activity (CDA). CDA is the contralateral negativity during the memory period of VWM experiments and reflects to the number of items in the memory array. CDA amplitude increases as number of items increase up to individual’s VWM limit [17]. (2) VWM training gain experiments: VWM training was developed to expand memory capacity over a period of 12-week. (3) The training effects to untrained tasks were studied in VWM transfer benefit experiments. Subjects were divided into two groups, training and control groups, to evaluate the behavioral evidence and neural activity changes. The transfer benefits were calculated by comparing between pre-training and post-training.

## 2. Material and Methods

### 2.1. Ethical Statement

The experimental protocols were approved by the Louisiana Tech University’s Institutional Review Board Committee. Informed written consent was obtained before experiments began.

### 2.2. Participants

Three experiments were implemented in this study (VWM load, VWM training gains and VWM transfer benefits). There were total of fifteen healthy young participants (18-31 years of age, including five females and ten males) in the VWM load task (the first experiment), five of the subjects in the training gain study (the second experiment), and ten subjects in transfer benefit tasks (the third experiment; five people per training or control group). All participants had normal or corrected to normal eye sight. None of them had a reported history of neurological or psychological disorder. All of them had no prior experience with the computerized VWM training.

### 2.3. Stimuli and Procedure

Participants were seated in front of a 17-inch computer screen and given visual cues at a distance of 80 - 100 cm in a dark sound-attenuated room. All stimuli were presented on a white background (RGB = 255, 255, 255). They were instructed to respond to the VWM arrays (“change” or “no-change” tasks) by pressing a button on a response pad as quickly and accurately as possible in assigned tasks. All experiments were conducted between 9:00 am to12:30 pm. Each subject was given 20 min of practice time prior to starting the VWM load task in order to minimize the individual differences and effects.

**Figure 1:**
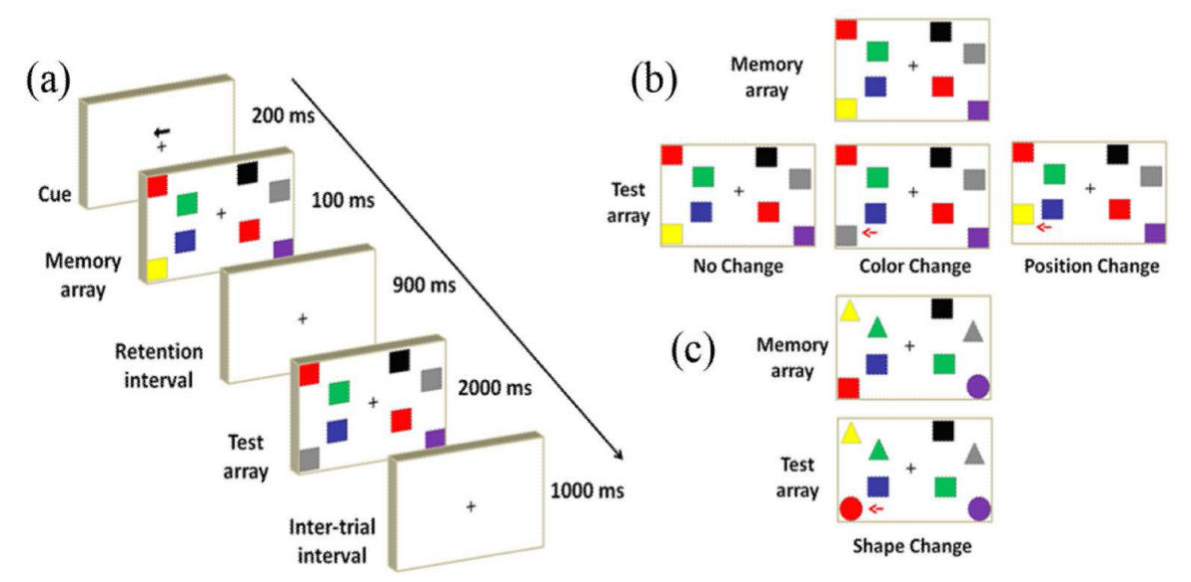
A schematic illustration of a visual working memory trial. (a) The sequence of each trial. (b) Three switch types (No-change or Change occurs in colors or positions) in the VWM load and training gain experiments. The red arrows indicate where the changes occur. (c)The fourth switch type in the transfer benefit experiments (No-change or Change in colors, positions or shapes).

The sequence of each trial is shown in Figure 1(a). At the beginning of each trial, a central arrow cue instructed the subjects to focus on either the left or right hemi-field for 200 ms. Memory arrays were displayed as 2, 4, or 6, colored squares (0.75 × 0.83°) consisting of 2-9 possible colors on each side of a central fixation cross for 100 ms. Different colors were used: red (RGB = 255, 0, 0), blue (RGB = 51, 51, 153), black (RGB = 0, 0, 0), yellow (RGB = 255, 255, 0), green (RGB = 0, 176, 80), purple (RGB = 112, 48, 160), gray (RGB = 128, 128, 128), orange (RGB = 255, 192, 0), and light blue (RGB = 0, 176, 240). The color of each square was randomly chosen one at a time (no repetitive color appeared in the same memory array on each side). These positions of colored squares were also randomly arranged in each trial. Fifty percent of the trials had the same colored and oriented squares in both memory and test arrays. The rest of the trials presented different targets in the test array. Each memory and test array pair was separated by a 900 ms retention interval. The test array would last, at most, 2000 ms, or until the subject responded. A 500-ms inter-trial interval would directly follow the termination of the test array. Figure 1(b) demonstrates three switch types in the VWM load experiment (the first experiment). VWM load experiment contained 2, 4, and 6 items on each side in the memory array and task difficulty levels were displayed from easy to complex conditions (2 to 6 items) in order to adapt the visual stimuli for subjects.

In the training gain experiment (the second experiment), participants completed 2 hours of VWM training one day per week in total of 12 weeks and the entire training time was 24 hours. The difference between the previous and the training gain experiments was that 8 colored items were included in the memory array to raise task difficulties. All experiments were recorded to observe the performance changes weekly. The transfer benefit task (the third experiment) not only required the observers to memorize colors and positions in the memory arrays, but also the shape of a particular item displayed in the test array (change-detection: No-change or change occur in color, position, or shape) is shown in Figure 1(c). The transfer benefit data from participants in the training group before and after the 12-week training session were labeled as TB1 and TB2, respectively. For comparison, the control group was assigned to participate only at the beginning and at the end of a 12-week period, without any training sessions in between. In order to reduce user fatigue, the recording sessions were separated into blocks with 5 min breaks. Depending on the experiment, each block consisted of 100 trials of VWM load experiment, 150 trials for training gain, or 150 trials for transfer benefit experiments. Consequently, the VWM load task was divided into 6 blocks, the training gain and transfer benefit tasks were separated into 4 blocks, with each block lasting approximately 10 min. This procedure resulted in total 600 trials per recording session. The experiment time required for each day was approximately 2 hr.

### 2.4. Electroencephalogram Recording

Electroencephalogram (EEG) signals were recorded using Net Amps 300 that connects a high-density 128 channel HydroCel Geodesic Sensor Net [18] (Electrical Geodesics Inc., Eugene, OR) with Net-Station 4.3 software. All electrode impedances were below 50 KΩ before recording was started [19]. Regions of interest (ROIs) around the parietal-occipital cortex were selected from the following standard international 10/20 posterior parietal electrodes for PO3 and PO4. Electrodes were referenced to the average Cz channel. All signals were anti-aliasing, low-pass filtered at 100 Hz, and digitized at a sample rate of 250 Hz.

### 2.5. EEG Preprocessing and Artifact Removal

The EEG data were filtered between 0.1 - 30 Hz. The data were segmented into individual conditions based on the number of items in the memory array. Each data segment, or epoch, lasted 1200 ms, and consisted of a 200 ms pre-memory array, a 100 ms memory array, and a 900 ms retention interval. The data were re-referenced to the average signal across all 128 electrodes. Bad channel replacement and bad trial elimination were applied to the data. The first 200 ms of each trial was used for baseline correction. The artifacts of poor skin contact, eye blink, eye movement, or muscle movement were detected and removed using independent component analysis (ICA) with extended Infomax algorithm [20] in EEGLAB [21]. Artifacts related ICs were removed and the remaining ICs were projected back to scalp for further ERP analysis. The details of ICA denoising approach can be seen by other studies [22]-[24].

### 2.6. Event-related Potential (ERP)

EEG waveforms were selected from parietal-occipital cortex area. Each condition, ERPs were obtained by averaged trials across participants. CDA (300-900 ms) was measured as the amplitude difference of ERPs between electrodes contralateral and ipsilateral to the location of the task-relevant cue in the memory array [25]. We acquired data from PO3/PO4, because the CDA amplitude was consistently high. However, the same patterns can be obtained over P3/P4, P5/P6, T5/T6, and O1/O2 electrode pairs [26].

### 2.7. Behavioral Measures and VWM Capacity

For each participant, the reaction time (RT) was calculated by the correctness of subject responses [27]. Incorrector no response trials were excluded from the analyses. Accuracies in percentage of correct responses ± standard error were calculated from all recorded trials. For VWM capacity, Pashler’s formula [28] [29] has been used to estimate the VWM capacity. $K=S\times \left(\frac{H-F}{1-F}\right)$, where K is the memory capacity, S is the number of memory array size, H is the hit rate, and F is the false alarm rate. Generally, WM capacity has been considered to be limited [30]. The assumption may assume that an observer can hold K index in memory from S items in the memory array, guided by the correct performance on VWM experiments. The commonly accepted capacity limit for an individual is 4-item [30]. Statistical analysis is done by one-way analysis of variance (ANOVA).

## 3. Results

### 3.1. VWM Load Experiment

The goal of VWM load experiments was to estimate the VWM capacity. Because the information that can be maintained and stored in memory is limited, it is important to understand the differences that may impact one’s ability to learn. Behavior and brain activity could predict the VWM capacity by task performance and the CDA amplitude of ERP [31] [32]. This VWM load experiment also included a parametric manipulation of the number of possible items in the memory array to further test the hypothesis that CDA amplitude can be used to understand VWM templates [26] [33]. The RT and CDA amplitude were measured at different levels of memory load.

#### 3.1.1. Behavior

**Figure 2:**
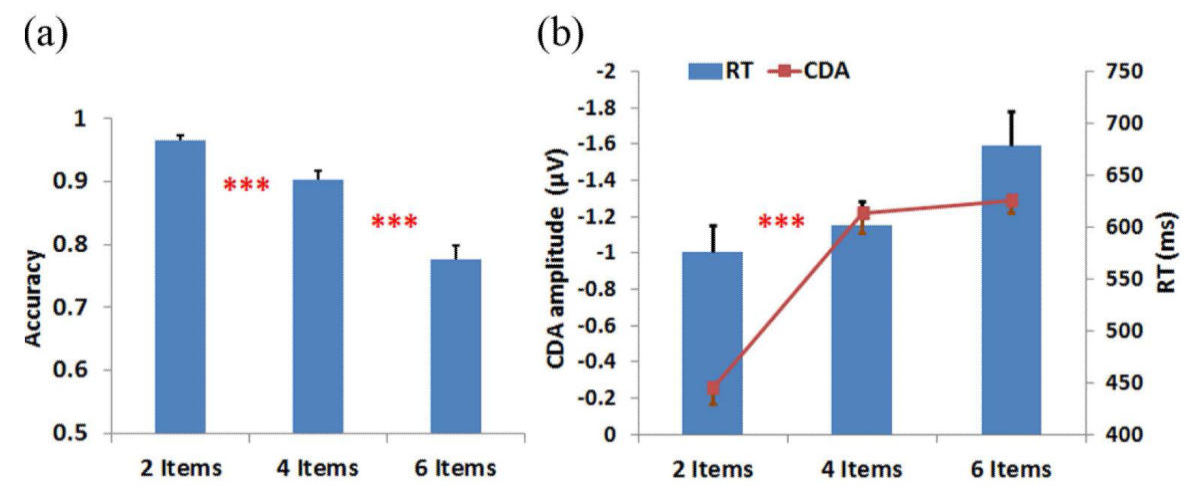
(a) The average accuracy across 15 participants in 2-, 4-, and 6-item conditions. (b) Combined response time (RT) and contralateral delay amplitude (CDA) results. Bar graph (right-axis) represents RT and line graph (left-axis) displays CDA amplitude. Error bars indicate the standard error in this study. ***p < 0.005 for comparisons between adjacent conditions in A and between mean CDA in B.

The average accuracy decreased significantly as the number of items increased (96.46 ± 0.85%, 90.27 ± 1.51%, 77.5 ± 2.35% for 2-, 4-, and 6-items, respectively; (F(1,29) = 12.69, p < 0.005; F(1,29) = 20.78, p < 0.005), as indicated in Figure 2(a). The large drop in accuracy for 6-item suggest that a memory array of this complexity may have exceeded the individual’s memory limit. The mean memory capacity was 3.25 items over 15 participants. In addition, the reaction time was significantly slower in 4- to 6-item conditions compared with 2-item (575.6 ± 25.6 ms vs. 602.0 ± 23.0 ms vs. 678.3 ± 28.1 ms, respectively; F(1,29) = 0.59, p > 0.05; F(1,29) = 4.42, p < 0.05). The reaction time was highly affected by the difficulty of memory array (Figure 2(b) bar graph).

#### 3.1.2. ERP

**Figure 3:**
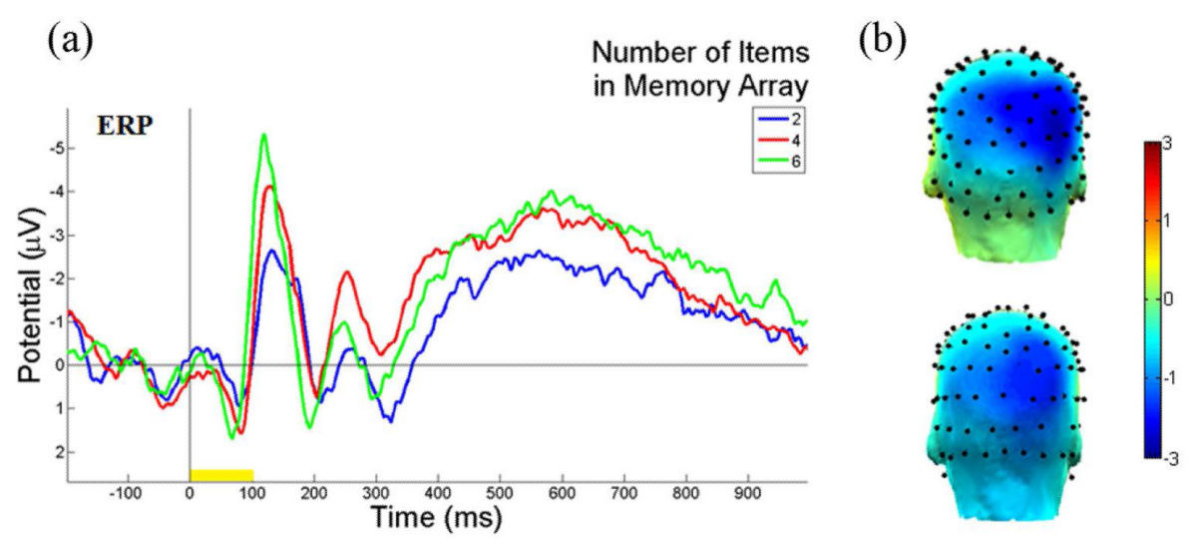
Illustrations of ERP contralateral effects of the VWM load experiment. (a) The average ERP waveforms of 15 subjects at PO4 electrode, contralateral to the location of the left cue during the stimulus encoding phase. The mean N1 (100 - 180 ms) component and duration 300 - 900 ms are where the contralateral ERP effects measured. The yellow bar (0-100 ms) on the timeline represents the epoch of the memory cue. (b) The 3D scalp maps of the VWM load experiments for 6-item at mean N1 (top) and the latency (300 - 900 ms) of mean ERP (bottom) at activated contralateral parietal-occipital regions.

**Figure 4:**
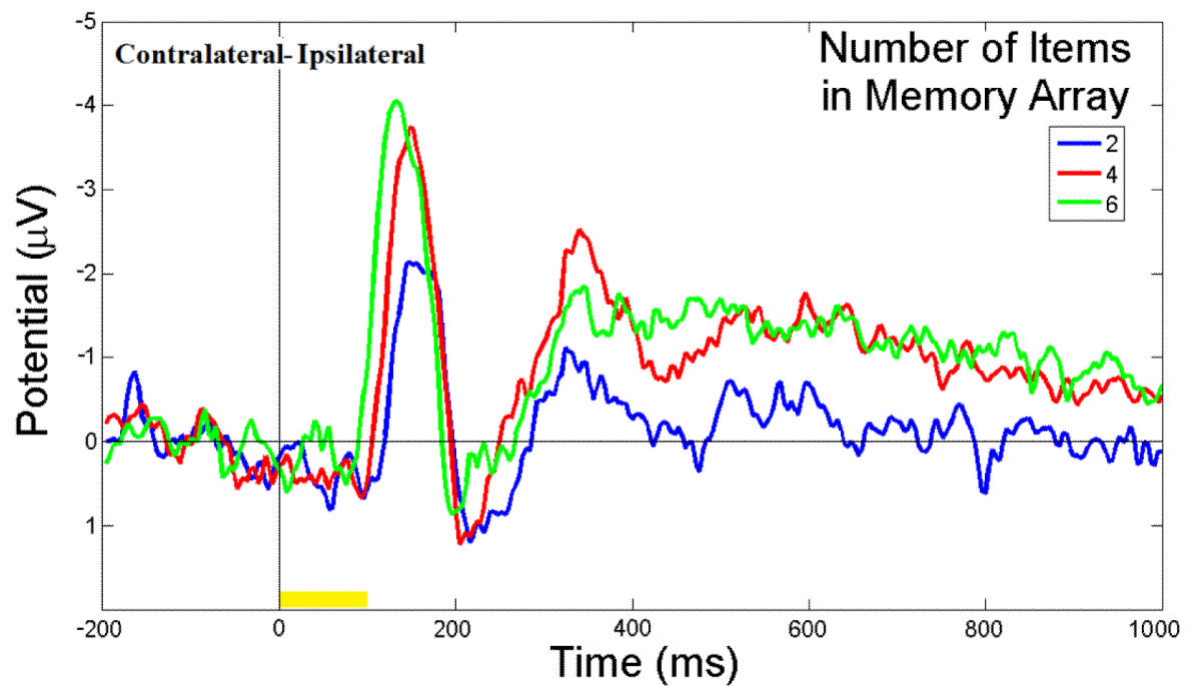
ERP difference waveforms of the VWM load experiment. The average ERP difference waveforms of 15 subjects between PO3 and PO4 electrodes during the stimulus encoding phase are shown. The ERP difference was measured between contralateral and ipsilateral waveforms to the location of the cue. The duration (300 - 900 ms after cue onset) was the CDA component measured. The yellow bar (0 - 100 ms) on the timeline represents the epoch of the memory cue.

Figure 3 illustrates the ERP contralateral effects for the VWM load experiment. The average ERP waveforms of the 15 subjects for 2-, 4-, and 6-item conditions at PO4 electrode contralateral to the location of the left cue during the stimulus encoding phase are illustrated. The mean N1 (100-180 ms) component and the duration of 300-900 ms were associated with the contralateral ERP effects measured in Figure 3(a). The 3D scalp topographies of the VWM load experiments for 6-item at mean N1 (top) and the latency (300-900 ms) of mean ERP (bottom) demonstrate activated contralateral parietal-occipital regions (Figure 3(b)). Figure 4 shows the average ERP difference waveforms from the parietal-occipital electrodes for the VWM load experiments. The CDA components during the time duration 300 - 900 ms after the memory cue were measured. A paired comparison of the mean CDA showed a significant increase from the 2- to 4-item condition (−0.2586 μV and −1.2212 μV; F(1,29) = 6.6, p < 0.005), whereas no difference was found between 4- and 6-item conditions (−1.2212 μV and −1.2904 μV; F(1,29) = 2.7, p > 0.05). This data provided statistical evidence that the CDA reaches a plateau at approximately 4-item which was the suggested maximum memory capacity for most people [34].

### 3.2. VWM Training Gain Experiment

The goal of the VWM training gain experiment was to develop VWM training procedures that would lead to the improvement of VWM performance. The effects of time period were materialized and investigated by comparing the difference in training gains for various levels of difficult tasks and observing the memory capacity improvement over 12-week.

#### 3.2.1. Behavior

**Figure 5:**
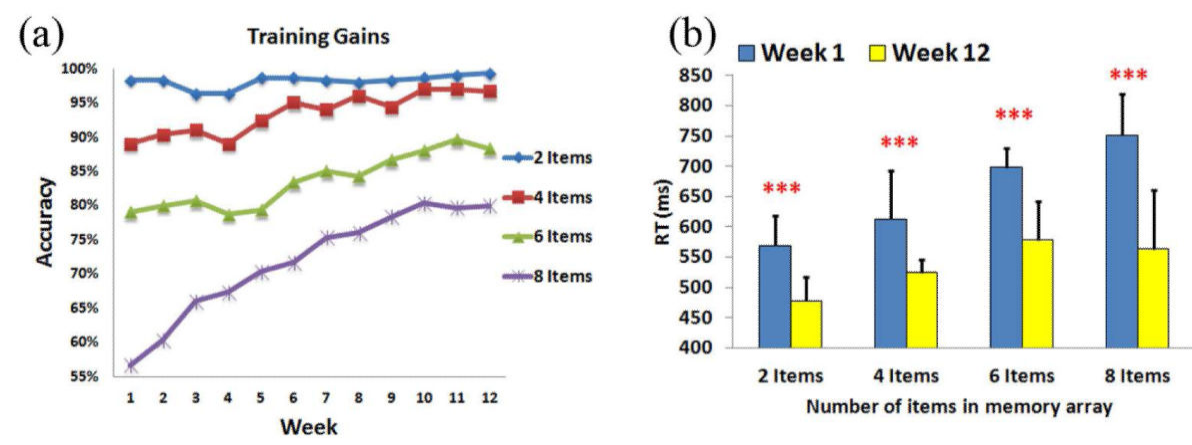
VWM training gain experiment results. (A) VWM training gains within the 12-week intervention period. The VWM performance improved week by week. (B) The comparison of RT results between the first and the last weeks. Subjects’ responses were faster in the 12th week than the first week in all conditions. ***p < 0.005.

VWM training gains within the 12-week intervention period are shown in Figure 5(a). At the beginning of the training gain tasks (1st week), the average accuracies of the 8-, and 6-item conditions were 56.67% and 79%, respectively. An accuracy exceeding 89% was found in the 4- and 2-item conditions. After 12 weeks of training, participants achieved significant improvements in the average accuracies (up to 80%, 88.33% and 96.67% for the 8-, 6- and 4-item trials, respectively). Because there was a high baseline level in the average accuracy (96%-99%) for the 2-item trials, no significant effect can be observed after 12-week of training. Particularly, the VWM performance may demonstrate an increasing trajectory from week to week. This result indicates that memory capacities of individuals have already shifted to the upper-limit level (index K ↑), which also indicates that the VWM capacities of the trainees have expanded through a long period of training. Consequently, Figure 5(b) shows that the reaction time has decreased after 12-weeks of training in all 2-item, 4-item, 6-item, and 8-item conditions (570 ms vs. 478 ms, 613 ms vs. 524 ms, 699 ms vs. 578 ms, and 751 ms vs. 563 ms; F(1,9) = 26.0, p < 0.005, F(1,9) = 29.3, p < 0.005, F(1,9) = 38.5, p < 0.005, F(1,9) = 69.3, p < 0.005, respectively), which suggests that the participants performed these tasks better in the last week than the beginning week.

### 3.3. VWM Transfer Benefit Experiment

The purpose of the VWM transfer benefit experiment was to study the impact of training gains on high-level cognitive VWM tasks (Change in color, position, and shape features). Subjects were separated into two groups, training and control groups, to evaluate the behavioral evidence and neural activity. Their performance on non-trained tasks was compared between pre-training (TB1) and post-training (TB2) sessions. The neural activity (TB2-TB1) that refers to changes of ERP amplitude can be used as a signal marker to illustrate the training effects.

#### 3.3.1. Behavior

**Figure 6:**
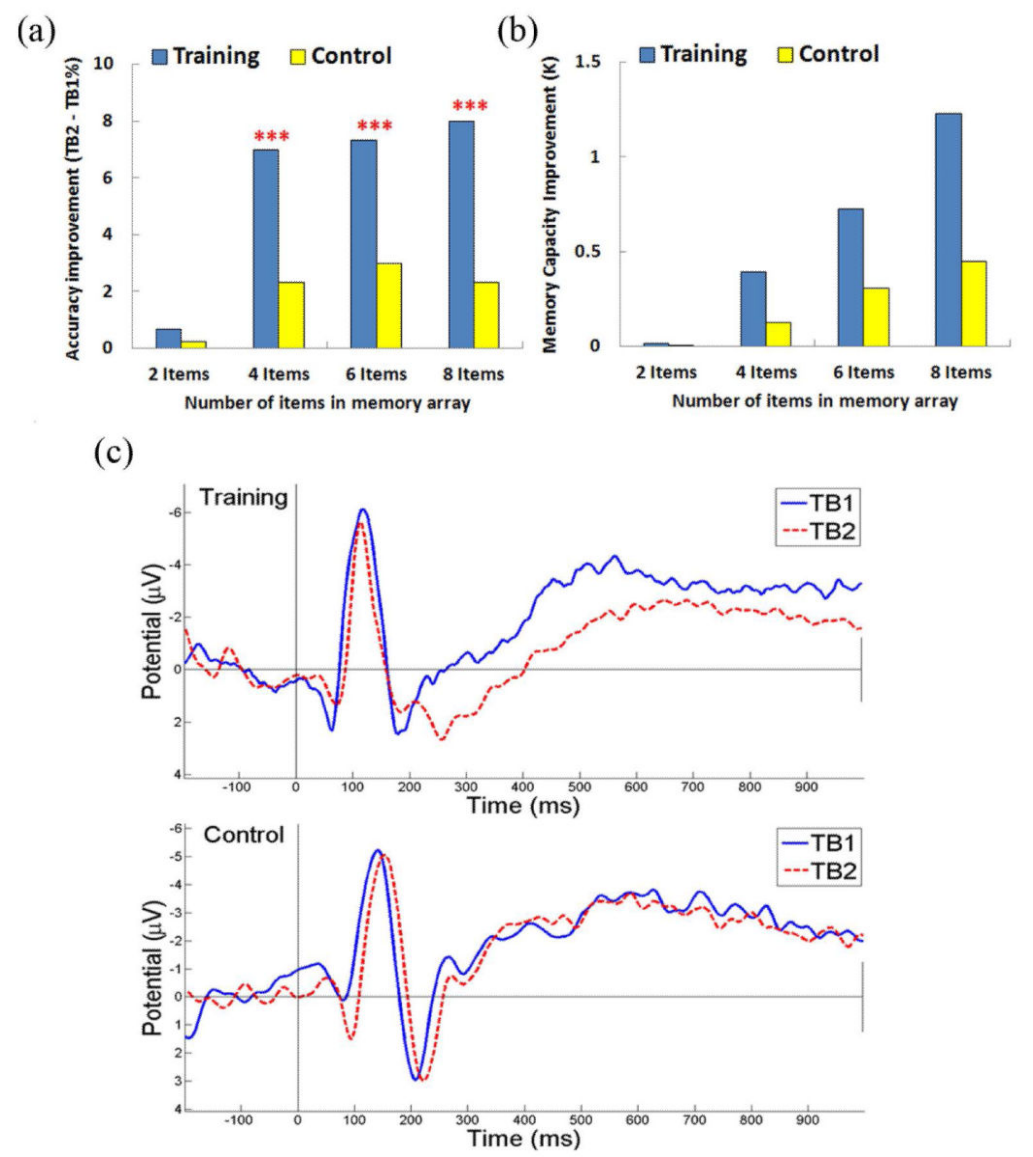
VWM transfer benefit experiment results. (A)VWM transfer benefit experiment between subjects with and without weekly training. The training group achieved a significant improvement over the control group. (B) The improvements of VWM capacity for training and control group (C) ERP of TB1/TB2 for training and control groups. Signals from parietal-occipital (ROIs) have significantly diminished amplitude (300 - 900 ms) for the training group at TB2, but not for the control group. *** p < 0.005.

To match the cognitive abilities and minimal variables, we selected subjects in the training and control groups who had similar performances from first experiment in 2-item, 4-item, and 6-item (97.25% vs. 94.5%, 88.13% vs. 95.13%, 81.25% vs. 81.5 %, respectively). The average accuracy improvement of the training group was significantly higher than the control group in 4-item, 6-item, and 8-item (7% vs. 2.33%, 7.33% vs. 3%, and 8% vs. 2.33%, respectively; F(1,9) = 23.6, p < 0.005, F(1,9) = 14.6, p < 0.005, F(1,9) = 21.1, p < 0.005, respectively). However, no significant difference for 2-item was found (0.67% vs. 0.25%; F(1,9) = 0.7, p > 0.05) (Figure 6(a)).The improvement of VWM capacity was estimated in Figure 6(b). The differences of VWM capacity improvement between training and control group in 2-item, 4-item, 6-item, and 8-item conditions of K were 0.009, 0.266, 0.42, and 0.78, respectively, which apparently demonstrate the significance of the improvements.

#### 3.3.2. ERP

The ERP analysis of the VWM transfer benefit was applied to evaluate the 8-item condition, because this highest difficult-level task show the greatest improvement (TB2 - TB1: 8%) for the trainees. In the training group, ERP(300 – 900 ms) amplitude was significantly different from TB1 to TB2 as shown in Figure 6(c) (−2.91 μV vs. −1.52 μV; F(1,9) = 8.42, p < 0.05). However, there was no significant difference for the control group (−3.5 μV vs. −3.06 μV; F(1,9) = 0.006, p > 0.05). This diminished ERP in the training group could be used as a predictor of VWM training effects on non-trained tasks. Recent reports have implicated that training could enhance VWM capacity and attention over time and also increase the brain activities in the prefrontal and parietal-occipital cortex [6] [14]. We analyzed the transfer benefits by proposing a subtractive measure where the difference between the post-training TB2 and the pre-training TB1 was computed for both training and control groups. The positive increase in neural activity was generated by training. For the control group, no significant change in ERP activity was observed.

## 4. Discussion

This study demonstrates the long-term training effects of VWM. In summary, we found that VWM capacity can be estimated based on accuracy and CDA level. First of all, neural evidence for VWM capacity limit, which approximates 4 discrete representations “magic number 4” [30], is observed in CDA waveforms. In particular, the average capacity across 15 participants is 3.25 items, which is common and similar to other studies [31] [35] [36]. Next, in Experiment 2, we are particularly interested in a longer training period (up to 12 weeks) than other studies (5 weeks) [15] in order to enhance training effects that indeed raise the number of objects stored in VWM. Obviously, the reaction time is shorter after 12-week of training in all 2-, 4-, 6-, and 8-item conditions, which suggests that the participants may feel more comfortable performing tasks at the end of training than the first week. Thus, VWM capacity can be improved through training over an extended period of time. Furthermore, in transfer benefit experiment, we also found that trainees have higher accuracy and memory capacity improvement than the control group. Explicitly, the increased neural activity is obtained in a subtractive measure (TB2 - TB1), which is the difference between post-training and pre-training for trainees. The training related changes of VWM capacity in K display the meaningfulness of the improvements, so the diminished ERP (300 - 900 ms) can be used as a neural marker of VWM training effects on non-trained tasks where ERP waveform changes led to improve memory capacity and VWM performance. Therefore, these cognitive improved measurements are important to reveal the strength of the training effects on non-trained tasks.

Because VWM plays a critical role in change-detection, especially when the memory array becomes more complex, it becomes more demanding as the memory array becomes more difficult [2]. In general, low-item condition results in less information, higher efficiency, higher accuracy, and low CDA amplitude. On the other hand, when many items are in a memory array, the subjects’ recall complex information is less efficient, resulting in a lower accuracy and higher CDA amplitude. Furthermore, individuals with low memory capacity depend on more working memory to perform VWM tasks. In contrast, participants with high memory capacity could perform VWM tasks much more easily and efficiently. Likewise, they are able to process and store more information during VWM experiments. Hence, this arrangement is supported by the accuracy, reaction time, VWM capacity, and CDA in VWM load experiments, representing that high-capacity individuals are more accurate and efficient under more complex conditions than low-capacity individuals. We hypothesize that those individuals with high VWM capacities can concentrate and manage distractions more effectively.

The results of this study clearly demonstrate that CDA component can be used to analyze the individual’s VWM capacity. Because CDA increases until it reaches a plateau at the individual’s limit, it can be observed for many types of WM studies, and the CDA pattern in the difference of ERP waveforms (Figure 4) has been consistent with other’s findings in which the CDA amplitude from 2-object searching is twice as high as for a single target [26]. Meanwhile, the CDA has disappeared when the subjects would repeat the same searching tasks after a short period of time [37]. This might be due to the fact that the participants’ VWM capacities are enhanced by training, or changed by the transition from a short-term memory to a long-term memory.

In addition, the present study also focuses on differences between trainees and controls in the transfer benefit experiment where the subjects would benefit from the training and then transfer to other tasks, such as near and far transfer experiments. In this study, we are mainly interested in the near transfer tasks based on the task similarity, whereas the far transfer tasks with major feature differences along with a non-adaptive control group of the visual-detect-training condition will be addressed in our future report [38] [39]. Because training gains and transfer effects can maintain across the 12-week intervention period, and the subtractive measure in transfer benefits suggests that neural activity induced by training often appears in VWM and attention [40], this study may assist cognitive decline and memory deficits due to normal aging by training and medication [41]. Moreover, the comparison between young and old adults would help understanding the training gains and transfer effects in systematic development [8] [42] [43], though we can expect that there are significant training gains for young adults in the most of VWM training paradigms.

Additionally, our method may be extended on a useful tool to predict an age-related trend in memory capacity through large cohorts of participants and the machine learning technique [44]. We are also interested in investigating how practice and training may impact an individual’s performance with aging [45], and the efficiencies of various training programs for different age groups. Besides, integrating EEG and transcranial magnetic stimulation (TMS) or transcranial direct-current stimulation (TDCS) to understand the influence of TMS/TDCS training is also important [46]. Especially, studies on VWM raise many unanswered questions, such as the optimal duration period and the sufficient amount of training time. Likewise, the comparison between visual WM and verbal WM tasks will be necessary to improve WM method development. We believe that the plasticity of the brain enables it to become more effective in memory, attention, processing information, thinking innovation, and solving problems [47] [48] through effective novel brain training simulations [49].

Interestingly, working memory tasks have also been used to study schizophrenia patients where prefrontal inefficiency and cognitive deficits have been found [7]. At the same time, recent studies have suggested that new neurogenesis is ongoing throughout life, so training and cognitive exercises may have a positive impact on building up strong neural connections and creating new brain networks [14] [50]. In principle, neural mechanisms of VWM can be simplified by the recurrent feedback loop to explain the neural activity network during VWM retention interval [51]. The recurrent feedback loop is easy to maintain a simple object, but difficult to keep representative complex object, which requires the interaction of more neurons. Neural oscillations are the driver that involves both local and global communication networks to maintain the recurrent activation and represent different objects. Therefore, prefrontal and parietal-occipital functional network could be a key to understand neural mechanisms of VWM [52].

The EEG based VWM approach is a precise and sensitive method to directly detect the neural activity changes from parietal-occipital electrodes [53]. Likewise, compared to other neuroimaging methods [54] [55], such as functional magnetic resonance image (fMRI) [56], EEG is less expensive, less time consuming and easier to operate. Another advance is that EEG is capable to resolve neural dynamic changes of integrated cognitive activity over milliseconds, which helps to further understand the short-term memory representation, whereas the fMRI requires the resolution over few seconds [57]. Overall, these results support our hypothesis that participants can benefit from training gains, and demonstrate sustained impacts are present on VWM capacity over a long period of time regarding a specific variety of tasks. Moreover, the general cognitive improvement associated with training needs further investigations.

## References

[1] Jonides J, Lewis RL (2008). The Mind and Brain of Short-Term Memory. Annual Review of Psychology.

[2] Luria R, Vogel EK (2011). Visual Search Demands Dictate Reliance on Working Memory Storage. Journal of Neuroscience.

[3] Brehmer Y, Westerberg H, Backman L (2012). Working-Memory Training in Younger and Older Adults: Training Gains, Transfer, and Maintenance. Frontiers in Human Neuroscience.

[4] Jeneson A, Wixted JT, Hopkins RO, Squire LR (2012). Visual Working Memory Capacity and the Medial Temporal Lobe. Journal of Neuroscience.

[5] Van Ewijk H, Heslenfeld DJ (2013). Visuospatial Working Memory in ADHD Patients, Unaffected Siblings, and Healthy Controls. Journal of Attention Disorders.

[6] Haut KM, Lim KO, MacDonald A (2010). Prefrontal Cortical Changes Following Cognitive Training in Patients with Chronic Schizophrenia: Effects of Practice, Generalization, and Specificity. Neuropsychopharmacology.

[7] Deserno L, Sterzer P, Wustenberg T, Heinz A, Schlagenhauf F (2012). Reduced Prefrontal-Parietal Effective Connectivity and Working Memory Deficits in Schizophrenia. Journal of Neuroscience.

[8] Zinke K, Zeintl M, Eschen A, Herzog C, Kliegel M (2012). Potentials and Limits of Plasticity Induced by Working Memory Training in Old-Old Age. Gerontology.

[9] Zhang C, Rodriguez C, Spaulding J, Aw TY, Feng J (2012). Age-Dependent and Tissue-Related Glutathione Redox Status in a Mouse Model of Alzheimer’s Disease. Journal of Alzheimer’s Disease.

[10] Zhang C, Rodriguez C, Circu ML, Aw TY, Feng J (2011). S-Glutathionyl Quantification in the Attomole Range Using Glutaredoxin-3-Catalyzed Cysteine Derivatization and Capillary Gel Electrophoresis with Laser-Induced Fluorescence Detection. Analytical and Bioanalytical Chemistry.

[11] Zhang C, Nestorova G, Rissman RA, Feng J (2013). Detection and Quantification of 8-hydroxy-2′-deoxyguanosine in Alzheimer’s Transgenic Mouse Urine Using Capillary Electrophoresis. Electrophoresis.

[12] Berry AS, Zanto TP (2010). The Influence of Perceptual Training on Working Memory in Older Adults. PLoS One.

[13] Gold JM, Wilk CM, McMahon RP, Buchanan RW, Luck SJ (2003). Working Memory for Visual Features and Conjunctions in Schizophrenia. Journal of Abnormal Psychology.

[14] Klingberg T (2010). Training and Plasticity of Working Memory. Trends in Cognitive Sciences.

[15] Olesen PJ, Westerberg H, Klingberg T (2004). Increased Prefrontal and Parietal Activity after Training of Working Memory. Nature Neuroscience.

[16] Brady TF, Konkle T, Alvarez GA (2011). A Review of Visual Memory Capacity: Beyond Individual Items and toward Structured Representations. Journal of Vision.

[17] Ikkai A, McCollough AW, Vogel EK (2010). Contralateral Delay Activity Provides a Neural Measure of the Number of Representations in Visual Working Memory. Journal of Neurophysiology.

[18] Tucker DM (1993). Spatial Sampling of Head Electrical Fields: The Geodesic Sensor Net. Electroencephalography and Clinical Neurophysiology.

[19] Ferree TC, Luu P, Russell GS, Tucker DM (2001). Scalp Electrode Impedance, Infection Risk, and EEG Data Quality. Clinical Neurophysiology.

[20] Jung TP, Makeig S, Westerfield M, Townsend J, Courchesne E (2000). Removal of Eye Activity Artifacts from Visual Event-Related Potentials in Normal and Clinical Subjects. Clinical Neurophysiology.

[21] Delorme A, Makeig S (2004). EEGLAB: An Open Source Toolbox for Analysis of Single-Trial EEG Dynamics Including Independent Component Analysis. Journal of Neuroscience Methods.

[22] Debener S, Ullsperger M, Siegel M, Fiehler K, von Cramon DY (2005). Trial-By-Trial Coupling of Concurrent Electroencephalogram and Functional Magnetic Resonance Imaging Identifies the Dynamics of Performance Monitoring. The Journal of Neuroscience.

[23] Kuo CC, Knight JL, Dressel CA, Chiu AW (2012). Non-Invasive BCI for the Decoding of Intended Arm Reaching Movement in Prosthetic Limb Control. American Journal of Biomedical Engineering.

[24] Kuo CC, Lin WS, Dressel CA, Chiu AW (2011). Classification of Intended Motor Movement Using Surface EEG Ensemble Empirical Mode Decomposition. http://dx.doi.org/10.1109/IEMBS.2011.6091550.

[25] Vogel EK, Machizawa MG (2004). Neural Activity Predicts Individual Differences in Visual Working Memory Capacity. Nature.

[26] Carlisle NB, Arita JT, Pardo D, Woodman GF (2011). Attentional Templates in Visual Working Memory. The Journal of Neuroscience.

[27] Itier RJ, Taylor MJ (2004). Effects of Repetition and Configural Changes on the Development of Face Recognition Processes. Developmental Science.

[28] Rouder JN, Morey RD, Morey CC, Cowan N (2011). How to Measure Working Memory Capacity in the Change Detection Paradigm. Psychonomic Bulletin & Review.

[29] Pashler H (1988). Familiarity and Visual Change Detection. Perception & Psychophysics.

[30] Cowan N (2001). The Magical Number 4 in Short-Term Memory: A Reconsideration of Mental Storage Capacity. Behavioral and Brain Sciences.

[31] Luck SJ, Vogel EK (1997). The Capacity of Visual Working Memory for Features and Conjunctions. Nature.

[32] Gevins A, Smith ME, McEvoy L, Yu D (1997). High-Resolution EEG Mapping of Cortical Activation Related to Working Memory: Effects of Task Difficulty, Type of Processing, and Practice. Cerebral Cortex.

[33] Pratt N, Willoughby A, Swick D (2011). Effects of Working Memory Load on Visual Selective Attention: Behavioral and Electrophysiological Evidence. Frontiers in Human Neuroscience.

[34] Fukuda K, Awh E, Vogel EK (2010). Discrete Capacity Limits in Visual Working Memory. Current Opinion in Neurobiology.

[35] Emrich SM, Al-Aidroos N, Pratt J, Ferber S (2009). Visual Search Elicits the Electrophysiological Marker of Visual Working Memory. PLoS ONE.

[36] Hollingworth A, Hwang S (2013). The Relationship between Visual Working Memory and Attention: Retention of Precise Colour Information in the Absence of Effects on Perceptual Selection. Philosophical Transactions of the Royal Society of London. Series B: Biological Sciences.

[37] Logan GD (2002). An Instance Theory of Attention and Memory. Psychological Review.

[38] Harrison TL, Shipstead Z, Hicks KL, Hambrick DZ, Redick TS (2013). Working Memory Training May Increase Working Memory Capacity but Not Fluid Intelligence. Psychological Science.

[39] Shipstead Z, Hicks KL, Engle RW (2012). Cogmed Working Memory Training: Does the Evidence Support the Claims?. Journal of Applied Research in Memory and Cognition.

[40] Rueda MR, Rothbart MK, McCandliss BD, Saccomanno L, Posner MI (2005). Training, Maturation, and Genetic Influences on the Development of Executive Attention. Proceedings of the National Academy of Sciences of the United States of America.

[41] Holmes J, Gathercole SE, Place M, Dunning AL, Hilton KA (2010). Working Memory Deficits Can Be Overcome: Impacts of Training and Medication on Working Memory in Children with ADHD. Applied Cognitive Psychology.

[42] Zehnder F, Martin M, Altgassen M, Clare L (2009). Memory Training Effects in Old Age as Markers of Plasticity: A Meta-Analysis. Restorative Neurology and Neuroscience.

[43] Richmond LL, Morrison AB, Chein JM, Olson IR (2011). Working Memory Training and Transfer in Older Adults. Psychology and Aging.

[44] Zhang C, Kuo CC, Chiu AW, Feng J (2012). Prediction of S-Glutathionylated Proteins Progression in Alzheimer’s Transgenic Mouse Model Using Principle Component Analysis. Journal of Alzheimer’s Disease.

[45] Gazzaley A, Clapp W, Kelley J, McEvoy K, Knight RT (2008). Age-Related Top-Down Suppression Deficit in the Early Stages of Cortical Visual Memory Processing. Proceedings of the National Academy of Sciences of the United States of America.

[46] Schulz H, Übelacker T, Keil J, Müller N, Weisz N (2013). Now I Am Ready—Now I Am Not: The Influence of Pre-TMS Oscillations and Corticomuscular Coherence on Motor-Evoked Potentials. Cerebral Cortex.

[47] Zanto TP, Rubens MT, Thangavel A, Gazzaley A (2011). Causal Role of the Prefrontal Cortex in Top-Down Modulation of Visual Processing and Working Memory. Nature Neuroscience.

[48] Clapp WC, Rubens MT, Sabharwal J, Gazzaley A (2011). Deficit in Switching between Functional Brain Networks Underlies the Impact of Multitasking on Working Memory in Older Adults. Proceedings of the National Academy of Sciences of the United States of America.

[49] Gates N, Valenzuela M (2010). Cognitive Exercise and Its Role in Cognitive Function in Older Adults. Current Psychiatry Reports.

[50] Palva JM, Monto S, Kulashekhar S, Palva S (2010). Neuronal Synchrony Reveals Working Memory Networks and Predicts Individual Memory Capacity. Proceedings of the National Academy of Sciences of the United States of America.

[51] Luck SJ, Vogel EK (2013). Visual Working Memory Capacity: From Psychophysics and Neurobiology to Individual Differences. Trends in Cognitive Science.

[52] Miller EK, Cohen JD (2001). An Integrative Theory of Prefrontal Cortex Function. Annual Review of Neuroscience.

[53] Barinaga M (1997). New Imaging Methods Provide a Better View into the Brain. Science.

[54] Dahlin E, Neely AS, Larsson A, Backman L, Nyberg L (2008). Transfer of Learning after Updating Training Mediated by the Striatum. Science.

[55] Moore CD, Cohen MX, Ranganath C (2006). Neural Mechanisms of Expert Skills in Visual Working Memory. The Journal of Neuroscience.

[56] Zimmer HD, Popp C, Reith W, Krick C (2012). Gains of Item-Specific Training in Visual Working Memory and Their Neural Correlates. Brain Research.

[57] Mitchell DJ, Cusack R (2011). The Temporal Evolution of Electromagnetic Markers Sensitive to the Capacity Limits of Visual Short-Term Memory. Frontiers in Human Neuroscience.

